# Three compartment bioimpedance spectroscopy in the nutritional assessment and the outcome of patients with advanced or end stage kidney disease: What have we learned so far?

**DOI:** 10.1111/hdi.12812

**Published:** 2020-01-22

**Authors:** Natascha J. H. Broers, Bernard Canaud, Marijke J. E. Dekker, Frank M. van der Sande, Stefano Stuard, Peter Wabel, Jeroen P. Kooman

**Affiliations:** ^1^ Department of Internal Medicine, Division of Nephrology Maastricht University Medical Center+ Maastricht The Netherlands; ^2^ NUTRIM School of Nutrition and Translational Research in Metabolism Maastricht University Maastricht The Netherlands; ^3^ Research and Development Fresenius Medical Care Deutschland GmbH Bad Homburg Germany

**Keywords:** Bioimpedance spectroscopy, body composition, chronic kidney disease, nutritional assessment

## Abstract

Bioimpedance spectroscopy (BIS) is an easily applicable tool to assess body composition. The three compartment model BIS (3C BIS) conventionally expresses body composition as lean tissue index (LTI) (lean tissue mass [LTM]/height in meters squared) and fat tissue index (FTI) (adipose tissue mass/height in meters squared), and a virtual compartment reflecting fluid overload (FO). It has been studied extensively in relation to diagnosis and treatment guidance of fluid status disorders in patients with advanced‐stage or end‐stage renal disease. It is the aim of this article to provide a narrative review on the relevance of 3C BIS in the nutritional assessment in this population. At a population level, LTI decreases after the start of hemodialysis, whereas FTI increases. LTI below the 10th percentile is a consistent predictor of outcome whereas a low FTI is predominantly associated with outcome when combined with a low LTI. Recent research also showed the connection between low LTI, inflammation, and FO, which are cumulatively associated with an increased mortality risk. However, studies toward nutritional interventions based on BIS data are still lacking in this population. In conclusion, 3C BIS, by disentangling the components of body mass index, has contributed to our understanding of the relevance of abnormalities in different body compartments in chronic kidney disease patients, and appears to be a valuable prognostic tool, at least at a population level. Studies assessing the effect of BIS guided nutritional intervention could further support its use in the daily clinical care for renal patients.

## INTRODUCTION

Protein energy wasting (PEW) is highly prevalent in dialysis patients, but also in patients with earlier stages of chronic kidney disease (CKD).^1^ The cause of PEW, which is an important risk factor for mortality, is multifactorial.^2^

The diagnosis of PEW in renal failure is according to current convention based on four different criteria, that is, body mass (low body weight, weight loss, or decreased total fat mass [FM]), decreased muscle mass, serum chemistry, and an estimation of dietary intake.^3^ The estimation of body fat and muscle mass as proposed in expert panels or guidelines is generally left to the discretion of the clinician, and can include different methods such as anthropometry, dual X‐ray absorptiometry (DEXA), and bioimpedance analysis (BIA).^3^, ^4^ BIA is an easily applicable and operator independent method which has a long history of research in end‐stage renal disease (ESRD). Whereas various BIA applications are available, such as single‐frequency, including vector based methods,^5^ whole body (or more appropriately called “wrist‐to‐ankle”) bioimpedance spectroscopy (BIS) appears at present to be most frequently studied.^6^, ^7^ Approximately one decade ago, a three compartment (3C) BIS model was introduced, which differentiates between three relevant compartments, that is, lean tissue mass (LTM), adipose tissue mass (ATM), and a calculated virtual entity reflecting fluid overload (FO), which is in the literature commonly described as the “overhydration” (OH) compartment,^8^ and which will be a negative volume in case of fluid depletion.

Whereas most research on 3C BIS has been devoted to abnormalities in fluid state,^6^, ^7^, ^9^ recent papers also have shed more light on its potential use in nutritional assessment. The aim of this short review is to discuss the available evidence on the use of the 3C BIS model and to explore the potential role in monitoring and management of PEW and other abnormalities in body composition in adult patients with advanced or end‐stage kidney failure.

## SHORT TECHNOLOGICAL BACKGROUND OF THE 3C BIS MODEL

**Figure 1:**
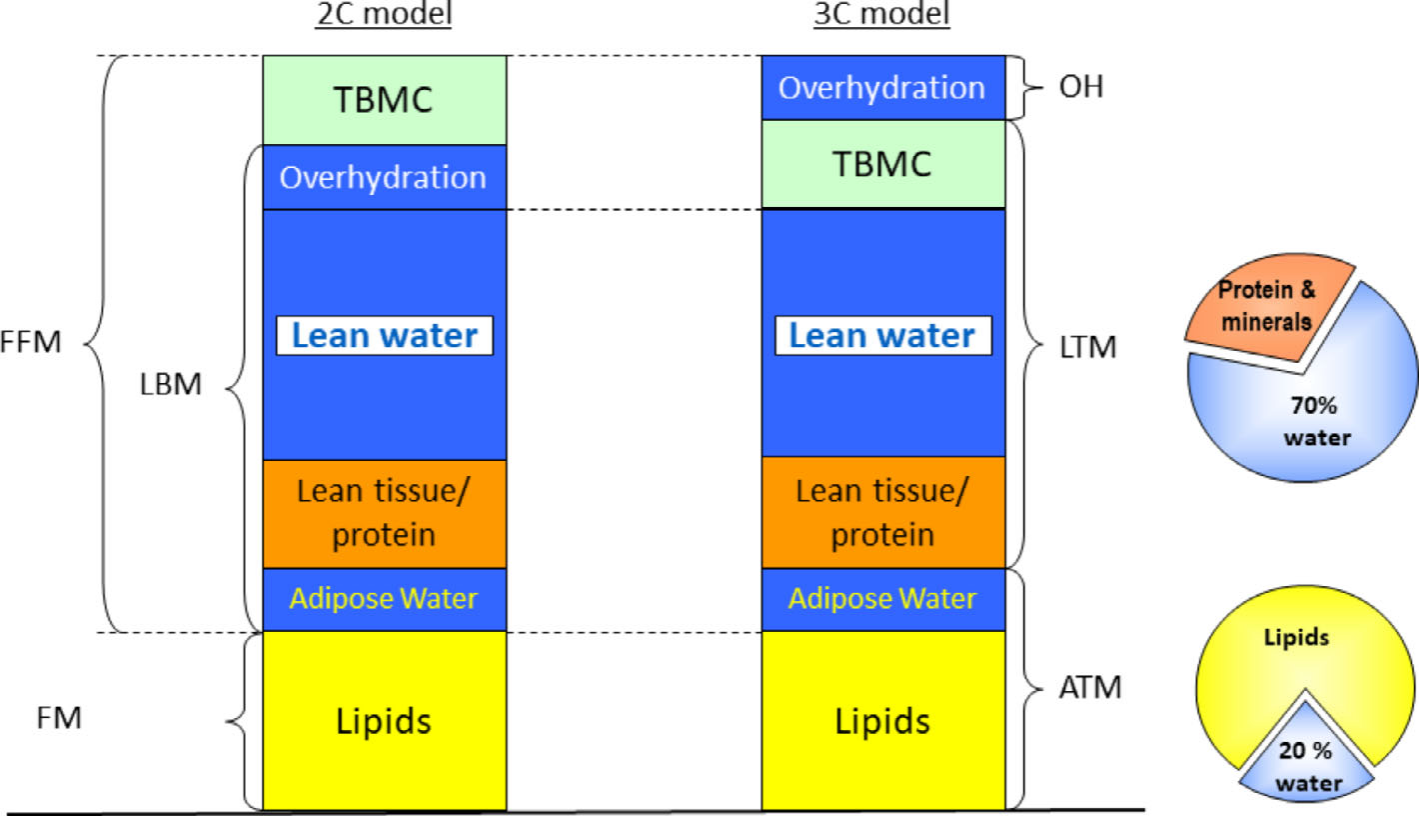
Distribution of body composition compartments 2C versus 3C model (by P. Wabel, Fresenius Medical Care D GmbH, Bad Homburg, Germany), as published by Broers et al.^14^ (reproduced with permission). 2C = two compartment; 3C = three compartment; ATM = adipose tissue mass; FFM = fat free mass; FM = fat mass; LBM = lean body mass; LTM = lean tissue mass; TBMC = total bone mineral content. [Color figure can be viewed at http://wileyonlinelibrary.com]

It is not the aim of this article to discuss the basics of BIS into detail, for which there are recent reviews available.^10^, ^11^ Basically, its principle is based on the measurement of the impedance of tissue on a broad range of frequencies of an alternating current. At low frequencies, the current only passes through the extracellular fluids (ECF), whereas at high frequencies the current also passes through the cell membranes. Complex models have been developed to use the variation in impedance with frequency to derive estimates of the ECF and intracellular fluids (ICF) based on the original Hanai model.^12^, ^13^ Differences between the two compartment (2C) models and the 3C models can be explained by different arrangements of body composition compartments, which are based on differences in hydration status of the compartments as is shown in Figure 1. The 2C model divides body composition compartments into a fat free mass (FFM) compartment and a FM compartment, where FFM is estimated based on the assumption that fat free tissue contains 73% of water.^15^ As the 2C model cannot distinguish excess volume due to fluid retention, predictions of FFM are influenced by the presence of FO.^14^, ^16^

The 3C model was developed based on validation with tracer dilution techniques, DEXA, and air displacement plethysmography in healthy controls and dialysis patients.^8^, ^12^ The 3C model recognizes the presence of ECF and ICF in adipose tissue and calculates the so‐called OH compartment based on the assumption of normal hydration ratios for lean and adipose tissue, respectively.^8^ Thus, the 3C model expresses three body compartments: LTM, ATM, and the OH compartment as an indicator of FO^8^ (Figure 1), which can also be negative and in this case points to fluid depletion.^7^, ^17^ Lipid containing FM can be derived from ATM by taking into account the hydration state of adipose tissue.^8^ In addition, LTM and ATM are usually normalized by dividing by height in meters squared, and by convention usually expressed in the literature as lean tissue index (LTI) and fat tissue index (FTI).^14^ The algorithm embedded in one of most cited multifrequency bioimpedance devices (i.e., Body Composition Monitor, BCM®), which applies the 3C method, has been assessed in different populations, mostly Caucasian,^12^ and validated against reference methods both for fluid volume and nutritional findings.^12^, ^18^ It should be recognized that BIS does not directly measure body composition, but only electrical properties of tissue which are used for the calculation of body compartments based on empirically derived values for tissue coefficients.^19^

## VALIDATION AGAINST REFERENCE METHODS

Comparison between body fluid compartments when assessed by tracer dilution techniques and BIS, in which a correction factor for body mass index (BMI) was included, have yielded acceptable limits of agreement (−0.4 ± 1.4 L [mean ± SD] for extracellular water [ECW] and 0.2 ± 2.0 L for intracellular water [ICW]).^12^ Volume estimation by BIS also seems to be reliable in patients with high BMI.^20^ However, it should be recognized that even different tracer dilution methods show disagreement between themselves in the assessment of fluid compartments which was comparable to the difference between tracer dilution methods and bioimpedance findings, and therefore the presence of real “gold standard” methods in this respect have been questioned.^21^ Using the 3C model, the mean difference in FM assessed between BIS and DEXA was 0.55 ± 3.3 kg in a Taiwanese population. The discrepancy between DEXA and BIS was not related to the degree of FO, but was more pronounced in patients with higher BMI, where BIS appeared to overestimate the fat compartment as compared to DEXA.^22^ In a study in 50 peritoneal dialysis (PD) patients, mean difference in estimated FM between 3C BIS and DEXA was 0.9 ± 5.7 kg (95% confidence interval [CI] = ‐10.5–12.3) and −0.3 ± 5.6 kg (95% CI = ‐11.8–10.8) for LTM.^23^ Relatively larger limits of agreement were also observed in the study of Zhou et al. in 120 patients with nondialysis dependent CKD.^24^ In order to facilitate the comparison, the authors calculated FFM by BIS by subtracting FM from body weight. FFM was higher in BIS as compared to DEXA by a mean difference of −2.8 kg (−12 to 6.5 kg), whereas mean FM was 3.1 kg lower when assessed by BIS as compared to DEXA (−6.8 to 13 kg).^24^ Importantly, in both studies, the discrepancy between DEXA and 3C BIS was related to the degree of FO, which has been shown to influence the estimation of body composition by DEXA.^16^ It is of importance to note that all of these studies have been performed in chronic disease population and none of them has been addressed to acutely ill patients.

In a study of 91 patients treated with online hemodiafiltration (OL‐HDF), 3C BIS was also compared between groups with or without PEW, as defined by the malnutrition‐inflammation score^25^ ≥ 5.^26^ In the PEW group, FTI but not LTI was lower as compared to the group without PEW. Notably, the prevalence of PEW was far lower (19.7%) as compared to that of LTI < 10th percentile, which was present in, respectively, 64.5% and 73.3% of patients with and without PEW.^26^

Data on the reproducibility of BIS in the nutritional assessment in renal patients are limited, but a study reported in abstract form showed a coefficient of variation of ECW and ICW in a time period of 30 days of 2.2% and 3.7%, respectively.^27^ However, although ECW and ICW are used for the calculation of body composition by 3C BIS, there are to the best of our knowledge no data yet on the reproducibility of FTI and LTI in the literature.

## PREVALENCE OF ABNORMALITIES IN BODY COMPOSITION IN DIALYSIS PATIENTS

A low LTI is highly prevalent in dialysis patients as compared with the LTI of healthy aged matched controls for which reference ranges are available.^28^ In the literature, cutoff limits of <10th or >90th percentile of an age‐matched and sex‐matched healthy population are usually used. In a study of Marcelli et al.^29^ in 37,345 European hemodialysis (HD) patients, LTI < 10th percentile was present in 44% of patients. LTI and FTI < 10th percentile was less prevalent, and only observed in 4.2% of patients, which means that in general, a low LTI is accompanied by a normal or increased FTI. Still, the combination of LTI < 10th percentile and FTI > 90th percentile, which would coincide with sarcopenic obesity, was only present in 3.5% of patients, whereas the combination of LTI < 10th percentile and FTI between the 10th and 90th percentile was observed in 39% of patients.^29^ The shift to higher BMI in dialysis patients appears to be due to an altered distribution between LTI and FTI in this population. For example, the group of patients with low LTI had, respectively, a mean BMI of 19 ± 1.9 kg/m^2^ when accompanied with FTI < 10th percentile, or a BMI of 25.4 ± 3.9 kg/m^2^ when accompanied with FTI between the 10th and 90th percentile. A normal body composition (e.g., both LTI and FTI between the 10th and 90th percentile) coincided with a mean BMI of 27.6 ± 4.0 kg/m^2^.^29^

The high presence of a low LTI appears to be a global phenomenon. In a study in Argentina in 934 patients, 58.8% of patients had LTI < 10th percentile.^30^

It has also been suggested that BIS could also play a role in the diagnosis of sarcopenic obesity in dialysis patients by investigating the relationship between ATM and LTM.^30^, ^31^

Changes in LTI and FTI or differences between groups appear to be detectable by BIS, at least on a population level. In a recent study, Marcelli et al. observed an increase in FTI of 1.0 kg/m^2^, and a decline in LTI of 0.4 kg/m^2^ in the first 2 years after the start of HD.^32^ These changes coincided with a mean increase in BMI of 0.6 kg/m^2^. These data were later confirmed by a study from Keane et al., who observed a mean increase in FM of 0.7 kg and a decline in LTM of 0.9 kg over a 2‐year period following the start of dialysis.^17^ In a cohort of 824 PD patients, LTI declined by a mean of 1.1 kg/m^2^ whereas FTI increased by 1.9 kg/ m^2^,^33^ with a significant inverse relationship between changes in both body compartments. These observations are in agreement with earlier data obtained by DEXA in a smaller group of dialysis patients.^34^ Another study found a lower LTI, but a comparable FTI in HD as compared with a matched cohort of PD patients.^35^

Interestingly, also seasonal differences in body composition in HD patients were detected by 3C BIS, with higher FM and lower LTM in the winter period.^36^

## RELATION BETWEEN ABNORMALITIES IN BODY COMPOSITION AND OUTCOME

**Figure 2:**
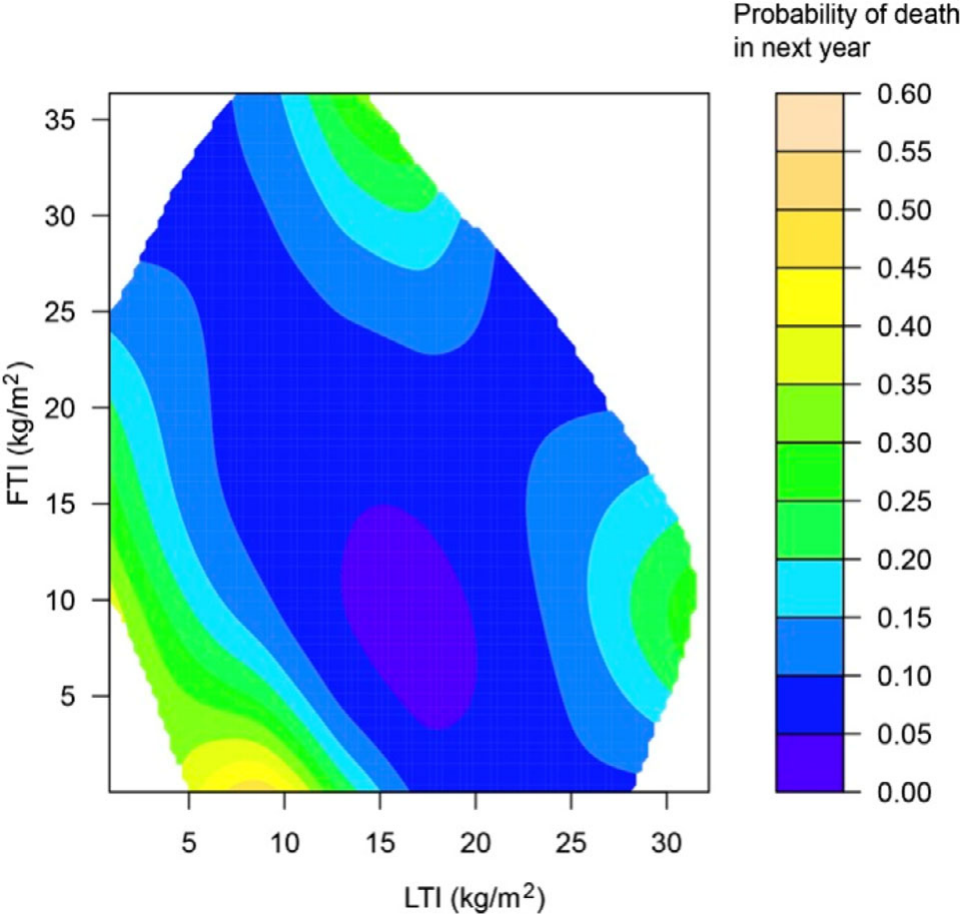
Interaction between LTI, FTI, and outcome in female HD patients by smoothing spline ANOVA (reproduced with permission from Marcelli et al.^32^). ANOVA = analysis of variance; FTI = fat tissue index; LTI = lean tissue index. [Color figure can be viewed at http://wileyonlinelibrary.com]

Previous research showed that especially low BMI in dialysis patients is associated with adverse outcome, whereas higher BMI is actually protective.^37^ However, as BMI is a composite parameter without the possibility to differentiate between LTM and ATM, there is a clear rationale for investigating the relation between specific body compartments and outcome, because targeted interventions may differ. Various studies have explored the relation between body composition assessed by 3C BIS and outcome. Rosenberger et al. observed a relation between LTI < 10th percentile and increased mortality in a study of 960 HD patients after adjustment for case‐mix.^38^ In a study including 697 Portuguese HD patients, a low FTI was associated with reduced survival.^39^ In a smaller group of HD patients, those with LTI < 10th percentile had a significantly higher risk of mortality,^40^ whereas in a cohort of 6395 Spanish HD patients, LTI below the 10th percentile was associated with increased mortality.^41^ In a study in 824 PD patients, both LTI below the 10th percentile and FTI above the 90th percentile were associated with increased mortality, although the latter relation lost significance after adjustment for C‐reactive protein (CRP) and serum albumin.^33^ In an international cohort study in 37,345 HD patients, both LTI < 10th percentile (hazard ratio [HR] = 1.53), and FTI < 10th percentile (HR = 1.19) were associated with significantly increased mortality as compared to LTI and FTI between the 10th and 90th percentile. The highest mortality (HR = 2.51) was observed in the group with a combination of both FTI and LTI < 10th percentile.^29^ Furthermore, the interaction between FTI/LTI and outcome was analyzed by means of smoothing spline analysis of variance in this study (Figure 2).

The relation between low LTI and outcome was confirmed in a meta‐analysis, in which LTI < 10th percentile was associated with mortality with a HR of 1.53.^42^

Also in patients with CKD stage 4–5, LTI appeared to be predictive for outcome. In a cohort of 356 patients, a lower LTI (defined as LTI < 14.1 kg/m^2^) was associated with an increased mortality during a mean follow‐up time of 22 months.^43^ These results were confirmed in a study in 326 patients with CKD stages 3–5, in whom LTI above the median value, but not high BMI or FTI, was associated with improved outcomes. Interestingly, the group with both LTI and FTI above the median showed the best outcomes.^44^

Whereas low FTI has been associated with adverse outcomes, as shown above, a very high FTI was associated with increased mortality as well. In an international study of our group,^29^ FTI > 90th percentile was associated with increased mortality without adjustment for LTI. However, in a combined model, those patients with LTI < 10th and FTI > 90th percentile had a lower mortality as compared to patients with a combination of LTI and FTI < 10th percentile, suggesting that high FTI in patients with significantly reduced LTI may actually be partially protective. Patients with normal LTI but FTI > 90th percentile also tended to have an increased mortality.^29^ Lee et al. observed a relation between the fat tissue/lean tissue (FM/LTM) ratio, as a proposed surrogate of sarcopenic obesity, and the risk of cardiac events and all‐cause mortality in a cohort of 130 HD patients.^31^ It should be noted that the quartile with the highest FM/LTM ratio was characterized both by the highest FM as well as the lowest LTM, which makes the interpretation of the individual contribution of the respective body compartments somewhat difficult to interpret.

Summarizing, a low LTI is associated with adverse outcomes, which also holds true for a low FTI in combination with a low LTI. This relation may be caused by the underlying disease state, leading to wasting, but also because skeletal muscle is an important reservoir of proteins.^42^, ^44^ Next to this, a low LTI contributes to muscle weakness and frailty with an increased risk of complications and may be associated with a reduced physical activity, which is at itself a risk factor for mortality.^31^, ^44^, ^45^ Moreover, also a low FTI may be a sign of serious underlying disorders, where severely depleted fat stores could interfere with the homeostatic response to a stressor such as infection or surgery, given the fact that fat is an important source of energy. In addition, the higher circulating lipoproteins associated with adipose tissue could provide prevention against endotoxins.^46^, ^47^, ^48^ On the other hand, visceral adiposity was related to an enhanced risk of cardiovascular complications in HD patients.^49^ Therefore, also in dialysis patients, extremes in body composition seem to be disadvantageous. A summary of articles reflecting body composition parameters in relation to outcome is presented in Table 1.

**Table 1:** Articles reflecting body composition parameters in relation to outcome

Group	Study population	Patient characteristics	Follow‐up period	Outcome
Rosenberger et al.^38^	HD (n = 748)* *complete cases out of n = 960	Age: 63 (54–73) y Male (%): 54	Median (IQR): 17 (10–33) mo	Diagnosed malnutrition (LTI < 10% of normal value) is an independent predictor of mortality. Mortality risk malnutrition vs. normal nutritional state: (HR = 1.66; 95% CI = 1.10–2.48, *P* = 0.015)*. * fully adjusted model
Caetano et al.^39^	HD (n = 697)	Age: 67 (55.5–76) y Male (%): 56.5	12 mo	Predictors of 1‐y all‐cause mortality*: Low FTI: (HR = 3.25; 95% CI = 1.33–7.96, *P* = 0.010). BMI < 18.5: (HR = 3.93; 95% CI = 1.99–7.74, *P* < 0.001) BMI = 25–29.9: (HR = 0.46; 95% CI = 0.23–0.92, *P* = 0.028). * fully adjusted model
Rymarz et al.^40^	HD (n = 48)	Age: 59.8 ± 15.5 y Male (%): 66.7	Mean ± SD: 29.93 ± 20.09 mo	Lower survival rate in patients with sarcopenia (defined as LTI < 10th percentile); not statistically significant (*P* = 0.055).
Castellano et al.^41^	HD* (n = 6395) *(Incident and prevalent patients)	Age: 67.6 ± 14.7 y Male (%): 62.7	Not defined; Study period: January 2012–December 2014	LTI < 10th percentile* carries higher relative risk of death. (OR = 1.57; 95% CI = 1.13–2.20, *P* < 0.05)**. * percentiles of LTI were calculated based on studied groups **multivariate regression.
Parthasarathy et al.^33^	PD (n = 824)	Age: 55.9 (47–68) y Male (%): 64	Up to 9 y	HR = 0.93; (0.86–1.00) for LTI > 10%, HR = 0.87 (0.78–0.97) for FTI < 90%. FTI lost significance after adjustment for biochemistry
Marcelli et al.^29^	HD (n = 37,345)	Age: 62.7 ± 15.2 y Male (%): 57	Median (25th–75th percentile): 266 (132–379) d	Both LTI and FTI within reference values of a healthy population indicate better survival in HD patients. All‐cause mortality risks fully adjusted models: HR only for LTI: Low LTI: (HR = 1.53; 95% CI = 1.40–1.66, *P* < 0.001). HRs only for FTI: Low FTI: (HR = 1.19; 95% CI = 1.08–1.31, *P* < 0.001) High FTI: (HR = 1.23; 95% CI = 1.02–1.47, *P* = 0.03). HRs for LTI + FTI combined: Low LTI + low FTI: (HR = 2.51; 95% CI = 2.12–2.96, *P* < 0.001) Low LTI + normal FTI: (HR = 1.63; 95% CI = 1.48–1.81, *P* < 0.001). Low LTI + high FTI: (HR = 1.74; 95% CI = 1.40–2.17, *P* < 0.001) Normal LTI + low FTI (HR = 1.42; 95% CI = 1.25–1.62, *P* < 0.001).
Hwang et al.^42^	HD (3 studies)			Meta‐analysis HR = 1.53 (1.41–1.66) for LTI < 10%
Vega et al.^43^	CKD4‐5 ND (n = 356)	Age: 67 ± 13 y Male (%): 64	Median (range): 22 (3–49) mo	Better survival in patients with high LTI. Survival analysis: (log‐rank, 9.47; *P* = 0.002). Independent relation between low LTI and mortality (*P* = 0.031, HR not showed*). Independent association cardiovascular mortality and low LTI. *multivariate regression.
Lee et al.^31^	HD (n = 131)	Age: 60.7 ± 13.6 y Male (%): 55.7	Mean ± SD: 53.1 ± 10.9 mo	The fat‐to‐lean (FM/LTM) mass ratio is an independent predictor of cardiac events and all‐cause mortality. Patients with high FM/LTM mass ratios had higher risks of cardiac events (*P* < 0.001 [log‐rank test]), and all‐cause death (*P* < 0.001 [log‐rank test]). Higher vs. lower FM/LTM ratio is a clinical indicator of all‐cause mortality (HR = 3.61; 95% CI = 1.07–12.13, *P* = 0.038*). * adjusted model

Age is given in mean ± standard deviation (SD) or median with interquartile range. BMI = body mass index; CI = confidence intervals; FTI = fat tissue index; HD = hemodialysis; HR = hazard ratio; IQR = interquartile range; LTI = lean tissue index; OR = odds ratio.

## ASSOCIATION OF ABNORMALITIES IN BODY COMPOSITION WITH OTHER RISK DOMAINS

Malnutrition often occurs in combination with abnormalities in other risk domains, most notably inflammation, and forms part of the so‐called Malnutrition Inflammation Atherosclerosis (MIA) syndrome.^50^ However, malnutrition also appears to be associated with abnormalities in fluid status. In a cohort of 338 patients with CKD stages 3–5, FO was inversely associated with LTI, and was incrementally associated with the MIA score,^51^ as well as with interleukin‐6.^52^ In a cohort of 478 patients with CKD stages 4 and 5, both LTI and FTI were lower in the fluid overloaded group, defined as an overhydration/extracellular volume (OH/ECV) ratio above 7%.^53^

Recently, we explored the association between LTM, inflammation, and FO. In a cohort of 8883 European prevalent dialysis patients, predialytic FO was more pronounced in patients with LTI < 10th percentile, and highest in the subgroup with the combined presence of LTI < 10th percentile and inflammation, defined as a high sensitive CRP (hsCRP) level above 6 mg/L.^54^ In 40% of this entire cohort, a low LTI was present in combination with either FO and/or inflammation, whereas in only 6.5% of patients, a low LTI was observed as an isolated phenomenon. Therefore, there are important arguments for a clustering of risk factors over different domains, which includes FO as an important novel risk factor. Furthermore, there also appears to be a clustering of abnormalities including those involved in the MIA syndrome with FO, although the relative contribution of abnormalities in the different risk domains might fluctuate between patients and over time.^7^, ^54^

Although in our study, the association between low LTI and mortality was highly apparent in combination with FO and/or inflammation, the association lost significance when corrected for other risk domains.^54^ However, in the study of Vega et al., a low LTI added independent prognostic information for risk of mortality,^43^ whereas in the study of Castellano et al., a low LTI remained predictive for mortality after adjustment for FO, serum albumin, and the Charlson comorbidity index.^41^ In a study in 529 PD patients, LTI was not significantly associated with outcome in a model adjusted for FO,^55^ whereas in another study in 824 patients, this relation remained significant after adjustment for FO.^33^

**Figure 3:**
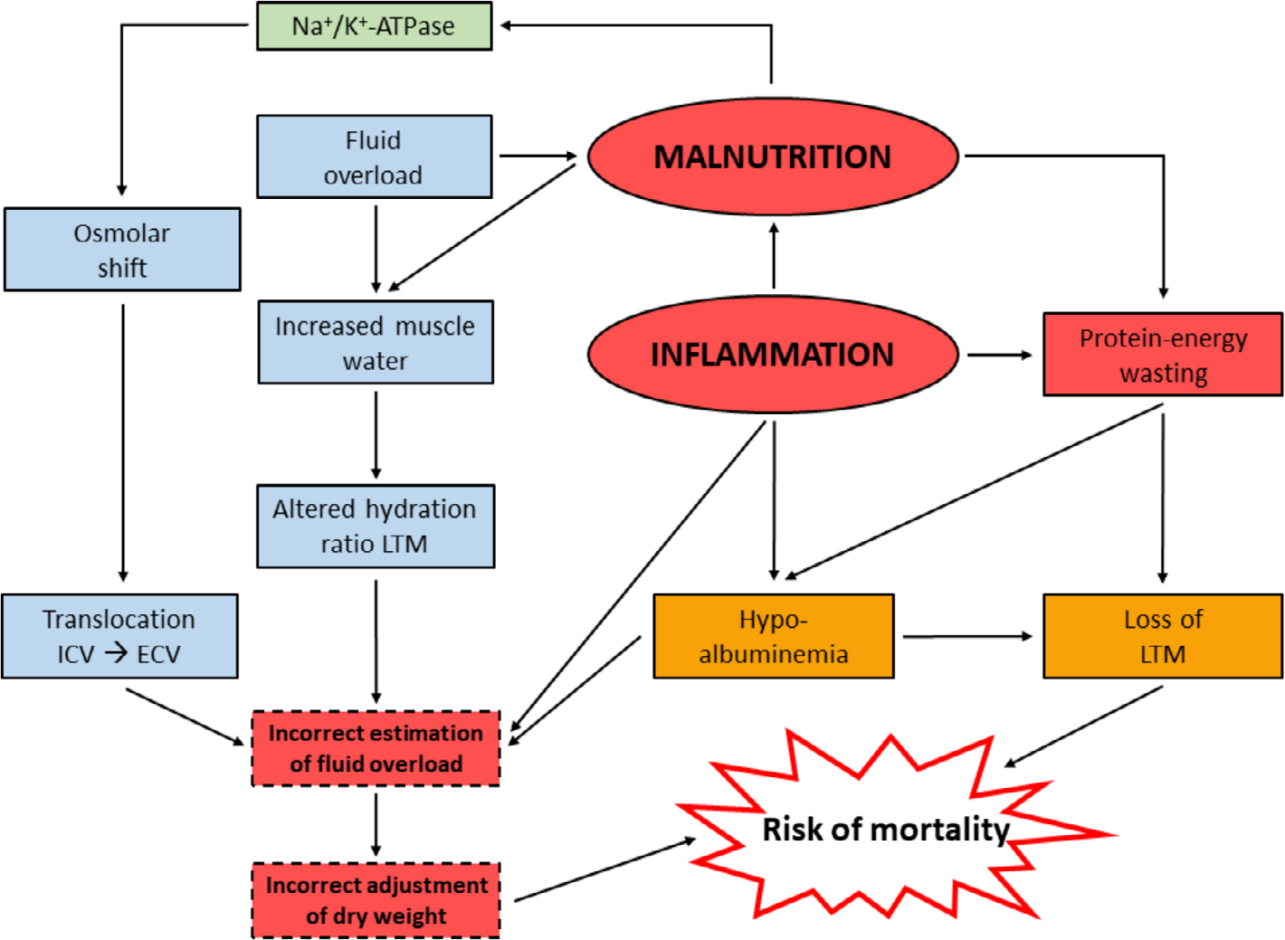
Hypothesized relation between malnutrition, fluid overload, and inflammation. ECV = extracellular volume; ICV = intracellular volume; LTM = lean tissue mass; Na+/K+‐ATPase = sodium‐potassium pump. [Color figure can be viewed at http://wileyonlinelibrary.com]

The mechanisms behind the relation between malnutrition, FO, and inflammation are likely complex (Figure 3) and may, in dialysis patients, include factors such as inflammation, hypoalbuminemia, and incorrect adjustment of target weight.^56^

However, whereas in malnourished patients without renal failure, ECV remained stable in an absolute sense, but increased relatively to body weight,^57^, ^58^ also in the absence of hypoalbuminemia. Given the fact that adipose tissue, which was also lower in these patients, contains ECW, actually a reduction in ECV might have been expected. The mechanisms for this relative expansion of ECV in nonuremic malnutrition, other than explained by a loss of intracellular mass per se^11^ remain unclear,^59^ although a translocation of ICW to ECW based on an osmotic shift from the intracellular to the extracellular space, or abnormalities of the Na^+^‐K^+^‐ATPase pump have been postulated.^60^, ^61^ Inhibition of the Na^+^‐K^+^‐ATPase pump may lead to cell shrinkage by secondary accumulation of intracellular Ca^2+^ resulting in a loss of amino acids and ions.^62^

Interestingly, relative water content of muscles was also found to be increased in nonuremic malnourished subjects, for reasons that have not been completely elucidated. Although the increase in relative water content of muscle, the major component of LTM, was relatively small (±3%), it violates to some degree the assumption of a fixed hydration level of LTM of 3C‐BIS which is used to calculate the OH compartment,^8^ which should therefore, in our opinion, be interpreted with some caution in patients with severe PEW.^26^ Keane et al. also found an increased OH (mean 1.1 L) in nonuremic malnourished patients.^63^ However, in nonuremic subjects, no difference in the hydration of LTM was observed between patients with mild and severe malnutrition.^64^ Also, a study by Chazot et al. showed a relation between brain natriuretic peptide and malnutrition (defined according to serum [pre]albumin and normalized protein nitrogen appearance),^65^ whereas Arias‐Guillen et al. observed a higher prevalence of FO in malnourished patients according to the clinical PEW criteria mentioned in the introduction of this article.^26^ This supports the FO‐malnutrition relationship also by the use of other methodologies than BIS.

Whereas a low LTI was related to FO and inflammation, the study of Lee et al. showed that in male subjects, the FM/LTM ratio was also positively related to hsCRP and interleukin‐6.^31^ In PD patients, changes in FTI were also positively related to changes in inflammatory status.^66^ This has mainly been attributed to the relation between visceral adiposity and systemic inflammation.^67^ However, a recent study suggested that the production of inflammatory cytokines was actually higher in subcutaneous as compared to visceral adipose tissue.^68^

A summary of articles reflecting abnormalities in body composition in relation with other risk domains in patients with advanced or end stage kidney disease is presented in Table 2.

**Table 2:** Abnormalities in body composition with other risk domains in patients with advanced or end stage kidney disease

Group	Study population	Patient characteristics	Outcome
Hung et al.^51^	CKD 3–5 (n = 338)	Age: 65.7 ± 13.5 y Male (%): 68.9	Negative correlation FO vs. LTI: *r* ^2^ = 0.038 Presence of MIA‐syndrome has an additive effect on the level of FO.
Wang et al.^52^	CKD 3–5 (n = 326)	Age: 65.8 ± 13.3 y Male (%): 68.7	Patients with low LTI (<10%) (n = 40) had significantly higher levels of interleukin‐6 (*P* = 0.017)
Tsai et al.^53^	CKD 4 and 5 (n = 478)	Age: 65.4 ± 12.7 y Male (%): 54.6	LTI and FTI are significantly lower in CKD patients with FO (hydration status > 7%), *P* = 0.003 and *P* = 0.01, respectively
Dekker et al.^54^	Prevalent HD (n = 8883)	Age: 63.5 ± 14.8 y Male (%): 57.2	Highest levels of predialysis FO (mean 3.06 L [95% CI = 2.79–3.34]) observed in patients with both LTI and FTI below <10th percentile + inflammation (hsCRP > 6 mg/L). Association between low LTI and mortality was highly apparent in combination with FO and/or inflammation (HR = 5.89 [95% CI = 4.28–8.10]) Low LTI was present in combination with either FO and/or inflammation in 40% of the population. Solely low LTI in 6.5% of the population
Vega et al.^43^	CKD 4 and 5 (n = 356)	Age: 67.0 ± 13.0 y Male (%): 64.0	LTI provides independent prognostic information for risk of mortality (*P* = 0.031)
Castellano et al.^41^	HD (n = 6395)	Age: 67.6 ± 14.7 y Male (%): 62.7	LTI is predictive for mortality after adjustment for FO, serum albumin, and the Charlson comorbidity index, where LTI < 10th percentile carries a higher relative risk of death (OR = 1.57; 95% CI = 1.13–2.20, *P* < 0.05)
O'Lone et al.^55^	PD (n = 529)	Age: 57.0 (46.7–68.8) y Male (%): 62.0	No significant associations between LTI to outcome in a model adjusted for FO
Parthasarathy et al.^33^	PD (n = 824)	Age: 55.9 (47.0–68.0) y Male (%): 64.0	Significant associations between LTI and mortality in a model adjusted for FO (HR = 0.88; 95% CI = 0.81–0.96)
Chazot et al.^65^	HD (n = 51)	Age: 65.3 ± 14.2 y Male (%): 54.9	Significantly increased levels of brain natriuretic peptide in patients with malnutrition
Arias‐Guillén et al.^26^	HD (n = 91)	Age: 60.0 ± 14.0 y Male (%): 29.7	Higher prevalence of FO in malnourished patients
Lee et al.^31^	HD (n = 131)	Age: 60.7 ± 13.6 y Male (%): 55.7	Significant associations between FM/LTM ratio vs. interleukin‐6 (*r* = 0.501), and hs‐CRP (*r* = 0.532) levels (*P* < 0.001)
Rincón Bello et al.^66^	Prevalent PD (n = 31)	Age: 57.4 ± 18.0 y Male (%): 45.2	Association between changes in FTI and changes in CRP (*r* = 0.382, *P* = 0.045)
Delgado et al.^67^	HD (n = 609)	Age: 56.1 ± 14.3 y Male (%): 57.0	Relation between visceral adiposity and systemic inflammation (CRP and interleukin‐6)

Age is given in mean ± standard deviation or median with interquartile range. CI = confidence intervals; CRP = C‐reactive protein; FO = fluid overload; FTI = fat tissue index; HD = hemodialysis; HR = hazard ratio; hs‐CRP = high sensitivity C‐reactive protein; LTI = lean tissue index; OR = odds ratio.

## INTERVENTIONAL STUDIES USING 3C BIS

Important is whether changes in body composition, measured by BIS, respond to nutritional intervention. Preliminary evidence suggests that this is indeed the case. In a nonrandomized study in which patients with a serum albumin level below 38 g/L received a high protein meal during dialysis, their FTI increased in contrast to a decline in FTI in the control group. However, LTI decreased both in the interventional and in the control groups.^69^ In addition, the PESET study used 3C BIS to monitor changes in body composition assigned to HD or OL‐HDF and observed a decline in LTM and an increase in ATM in HD as compared to the OL‐HDF patients.^70^ No study has yet prescribed nutritional intervention according to abnormalities in body composition by BIS, although recently a proposal was made for nutritional monitoring and intervention based on FTI and LTI criteria.^26^

## THE ROLE OF BIS IN THE INTEGRATED ASSESSMENT OF DIALYSIS PATIENTS

**Figure 4:**
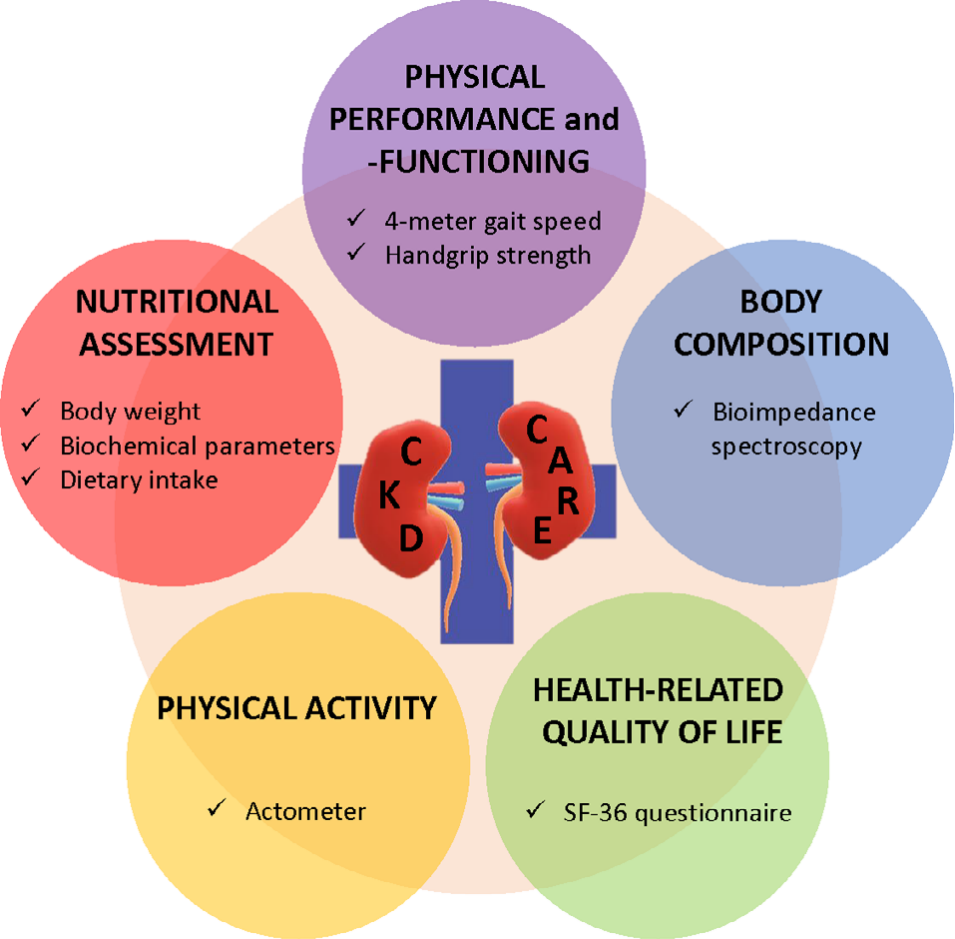
Proposed role of bioimpedance spectroscopy in the multidimensional assessment of nutritional and functional status in patients with advanced chronic kidney disease. SF‐36 = short form‐36. [Color figure can be viewed at http://wileyonlinelibrary.com]

It is important to realize that assessment of body composition is only part of the nutritional and functional assessment of dialysis patients. Earlier recommendations have suggested to include body composition as one of the criteria for PEW.^3^, ^71^ In addition, a study of Chen et al. already showed the added value of measuring LTI with the BCM as a screening tool for nutritional status, given the fact that low LTI as a marker of protein wasting, is seen as the primary component of PEW.^72^ It is of importance to realize that abnormalities in body composition are part of a wider, but only partly overlapping spectrum, including physical inactivity, a reduction in muscle strength, and a reduction in health‐related quality of life^14^, ^73^, ^74^, ^75^ in which the latter may surpass a reduction of muscle mass in the prediction of outcome.^76^ Indeed, recent guidelines on sarcopenia recommend to include combined assessments of muscle mass, muscle strength, and physical performance.^77^ Next to this, it would be rational to include physical activity in this assessment as it plays an important role as a determinant of muscle strength and the frailty syndrome.^78^, ^79^ Therefore, to our opinion BIS should not be implemented as a single solution, but as part of a multimodal and recurrent assessment strategy in which the different dimensions of PEW are incorporated, next to a parameter for muscle strength, physical activity, physical performance, and health‐related quality of life. Easily applicable tools such as handgrip strength measurements, actometers, the 4‐meter gait speed test, and short form‐36 (SF‐36) questionnaires can assess all of these domains (Figure 4). However, further research is recommended with regard to the implementation of BIS measurements for determination of fluid status and nutritional guidance of ESRD patients to show the effect of using the BCM on clinical outcomes in this patient group.^80^

## CONCLUSION

In the last years, assessment of body composition using the 3C BIS model has provided a wealth of information, not only regarding the importance of FO, but also that of other abnormalities in body composition in patients with ESRD and earlier stages of CKD. Its ease of use has led to the availability of large cohorts in which the relation between body composition and outcome can be studied. The use of 3C BIS has also facilitated the interpretation of the relation between outcome and the interaction of abnormalities in different body compartments. Despite the relatively wide limits of agreement with reference techniques such as DEXA found in some studies, the available studies consistently show that a low LTI is related to increased mortality, and as such has predictive validity. It has also been shown that the concomitant presence of a low FTI can add to the risk associated with low LTI. On the other hand, excessively high FTI may also carry a risk for especially cardiovascular mortality. The available information thus suggests that assessment of body composition by the 3C BIS model provides highly relevant prognostic information, at least at a population level.

To the best of our knowledge, in contrast to studies using BIS for the assessment of fluid status,^81^ studies assessing the effect of nutritional interventions are still limited. Studies showing the validity of BIS‐guided nutritional intervention would provide a strong rationale for the implementation of BIS in the prevention of PEW. At present, the main argument for its use in clinical practice is its usefulness in risk stratification, in combination with assessment of FO, which provides prognostic information about different risk domains using a single measurement. This besides a role in a holistic nutritional and functional assessment using easily applicable tools in a dialysis population.

## AUTHOR CONTRIBUTIONS

Manuscript draft: NJHB and JPK; Manuscript revision: BC, MJED, FMvdS, SS, PW, and JPK; Manuscript approval of final version: NJHB, BC, MJED, FMvdS, SS, PW, and JPK.
